# Leptin, Interleukin 6, and Vascular Endothelial Growth Factor as Potential Predictors of Primary Hypertension in Children and Adolescents with Obesity

**DOI:** 10.3390/ijms27020559

**Published:** 2026-01-06

**Authors:** Anna Sosnicka, Marta Jaskulak, Izabela Rysz, Malgorzata Grzybowska, Milena Deptuła, Małgorzata Zawrzykraj, Michał Pikuła, Iwona Ben-Skowronek, Katarzyna Zorena

**Affiliations:** 1 Department of Immunobiology and Environment Microbiology, Faculty of Health Sciences, Medical University of Gdansk, 80-210 Gdansk, Poland; anna.skowronek@gumed.edu.pl (A.S.); katarzyna.zorena@gumed.edu.pl (K.Z.); 2 Department of Pediatric Endocrinology and Diabetology, University Children’s Hospital, Medical University of Lublin, 20-093 Lublin, Poland; izabela.rysz@umlub.pl (I.R.); iwona.ben-skowronek@umlub.pl (I.B.-S.); 3 Laboratory of Tissue Engineering and Regenerative Medicine, Division of Embryology, Medical University of Gdansk, 80-210 Gdansk, Poland; milenadeptula@gumed.edu.pl (M.D.); michal.pikula@gumed.edu.pl (M.P.); 4 Laboratory of Tissue Engineering and Regenerative Medicine, Division of Clinical Anatomy, Medical University of Gdansk, 80-210 Gdansk, Poland; m.zawrzykraj@gumed.edu.pl; 5 Department of Biochemistry, University of Physical Education and Sport, 80-336 Gdansk, Poland

**Keywords:** children, hypertension, obesity, adipokines

## Abstract

The increasing prevalence of obesity-related primary arterial hypertension (PAH) in the pediatric population emphasizes the need to develop new biomarkers that can aid in clinical practice for prevention or early diagnosis of the cardiovascular disease. The objective of the present study was to evaluate the relationship between selected adipokines, cytokines, and blood pressure (BP) values in children with obesity. A total of 78 children participated in the study: 60 children with obesity (study group) and 18 children with normal weight (control group). Blood pressure was measured according to guidelines. Serum levels of metabolic and inflammatory markers, including leptin, adiponectin, resistin, ghrelin, interleukin 6 (IL-6), interleukin 10 (IL-10), tumor necrosis factor α (TNF-α), vascular endothelial growth factor (VEGF), and insulin were determined using multiplex immunoassays. Statistical analysis included correlation and ROC tests to identify potential predictors of PAH. The study group had significantly higher systolic and diastolic BP compared to the control group (*p* < 0.0001). Serum levels of leptin, IL-6, VEGF, insulin, and resistin were increased in the study group. Leptin, IL-6 and resistin correlated positively with BP values (*p* < 0.05), while ghrelin and adiponectin correlated negatively. ROC analysis identified leptin, IL-6, and VEGF as the most promising biomarkers for predicting PAH. The results confirm the role of adipokines and cytokines in the pathogenesis of PAH. The assessment of adipokine and cytokine profiles complements traditional anthropometric parameters such as BMI in assessing cardiovascular risk. Leptin, IL-6, and VEGF presented the strongest correlation with hypertension, suggesting their potential in future diagnostic and preventive strategies.

## 1. Introduction

In recent decades, arterial hypertension (AH) among children and adolescents under 19 years of age has become an increasingly common public health problem [1,2,3,4]. The incidence of this condition increases with age and affects approximately 3–5% of people aged 0 to 18. Interestingly, during puberty, there is a sharp increase in the incidence of primary arterial hypertension (PAH), which is more often diagnosed in boys than in girls. This phenomenon is associated with the physiological increase in blood pressure (BP) during puberty, especially in boys, in whom AH is diagnosed 3–4 times more often than in their peers [2,5].

PAH accounts for approximately 50% of all AH diagnoses in children and adolescents and is closely related to the spreading epidemic of overweight and obesity in the pediatric population. Moreover, obesity is among the main modifiable risk factors for hypertension, alongside smoking and excessive consumption of salt and energy drinks [1,3,6,7].

Abdominal obesity especially causes numerous metabolic and hormonal disorders that contribute to increased BP. The diagnosis itself and the implementation of treatment for hypertension at a young age should be based on a comprehensive analysis of patients, which consists of the assessment of anthropometric indicators, repeated measurements of BP in outpatient settings, and taking into account environmental and family factors [1,3,6].

The current literature increasingly emphasizes that the function of adipose tissue is not only passive energy storage, but also the release of bioactive metabolites, including adipokines and cytokines. They play a key role in the homeostasis of the body. Excessive pathological growth of adipose tissue leads to dysregulation of its endocrine function, which results in disruption of the aforementioned balance and can lead to numerous diseases, including the development of AH. The mechanisms responsible for the increase in BP include, among others, tissue resistance to insulin with secondary hyperinsulinism, excessive activation of the sympathetic nervous system, disorders in the renin–angiotensin–aldosterone system (RAA), and chronic low-intensity inflammation [1,6,8].

Although the number of studies focusing on metabolomics in the context of hypertension in children and adolescents with obesity is limited, the most important adipokines and cytokines affecting the cardiovascular system include adiponectin, leptin, and IL-6 [9,10]. The first of these is described as an adipokine that can have a protective effect on blood vessels, thus reducing the risk of developing AH. In the literature, its vasoprotective functions include inhibition of the transformation of macrophages into foam cells, inhibition of monocyte adhesion to endothelial cells, increasing nitric oxide synthesis, and stimulating angiogenesis. Adiponectin levels are reduced in people suffering from obesity, so blood vessels are more susceptible to adverse endothelial changes that underlie the pathogenesis of cardiovascular diseases (CVDs) [10,11,12,13,14]. Leptin, the satiety hormone, is produced by mature adipocytes and released into the bloodstream, where it is transported to the brain. Its concentration in serum increases with the development of obesity, but paradoxically, it does not reduce appetite, while simultaneously causing an increase in the activity of the sympathetic nervous system. This effect can lead to increased BP values [14,15,16,17,18,19,20]. Among the cytokines, interleukin-6 (IL-6) and vascular endothelial growth factor (VEGF) are of particular note. It is known that in obesity, they are associated with a low or medium level of inflammation, the mediators of which are, among others, the above-mentioned cytokines. An increase in their concentration in the body may promote changes in the structure of blood vessels, inflammatory processes, and, through an increase in vascular resistance, may result in the development of hypertension [21]. The role of resistin and ghrelin, which also belong to the adipokine family, remains ambiguous, but some studies suggest their potential influence on the regulation of vascular tone. Their mechanism of action seems to be similar to that of cytokines [22,23,24].

In clinical practice, more and more emphasis is being placed on preventing and early detection of AH and obesity, because the co-occurrence of these diseases in childhood and adolescence significantly increases the risk of developing organ complications. The most serious complications of hypertension in the course of obesity include left ventricular hypertrophy and kidney damage, both before and after the age of 18 [1,3,25].

In this study, an attempt was made to determine the relationship between the levels of selected adipokines and cytokines and blood pressure values in children and adolescents with obesity. Analysis of these parameters may allow for the identification of predictive biomarkers of hypertension, which in the future may contribute to more effective prevention and prediction of the risk of hypertension and a better understanding of the pathomechanisms of obesity associated with cardiovascular risk.

## 2. Results

The study included a total of 78 children and adolescents under the age of 18, including 60 individuals in the study group and 18 individuals in the control group. The percentage of boys and girls in the study group was 46.7% and 53.3%, respectively, while the control group had an equal number of girls and boys. There were no statistically significant differences in gender (*p* = 0.8038) and age (*p* = 0.0570) between the groups. The mean age of participants was 14.6 ± 1.5 years in the study group and 12.4 ± 3.8 years in the control group, respectively. The distribution of maturity phases in relation to the groups differed statistically significantly (*p* = 0.0351). In the study group, most children were in Tanner stages of puberty 3–5, with more than half of the subjects being in Tanner stage of puberty 5. In the control group, the majority of children were in Tanner stage 4, but the remaining subjects in this group were evenly distributed between Tanner stages 1, 2, 3, and 5. The average value of body weight in the study group was 101.3 (17.7) kg and in the control group 41.4 (15.6) kg, while the average value of height in the study group was 1.7 (0.1) m and in the control group 1.5 (0.2) m. Body weight and height were significantly higher in the study group (*p* < 0.0001 and *p* = 0.0039). The average value of BMI in the study group was 35.7 (4.6) kg/m^2^ and was significantly higher (*p* < 0.0001) than in the control group, in which it was 17.7 (2.6) kg/m^2^.

In the study and control groups, basic parameters concerning carbohydrate and lipid metabolism were assessed. The average values of glucose and high-density lipoprotein (HDL), 80.99 (8.13) mg/dL and 40.42 (10.72) mg/dL, respectively, were statistically significantly lower (*p* < 0.001 and *p* = 0.0002) in the study group than in the control group, where the average value of glucose was 93.29 (5.50) mg/dL and of HDL was 53.78 (11.86) mg/dL. At the same time, the average value of insulin, low-density lipoprotein (LDL) and triglycerides (TGs), 711.50 (737.31) pg/mL, 110.17 (29.79) mg/dL and 111.08 (46.62) mg/dL, respectively, in the study group were significantly higher (*p* = 0.0022, *p* = 0.0064 and *p* = 0.0378) than the average value of these parameters in the control group, which were estimated at the following levels: insulin, 277.83 (111.89) pg/mL; LDL, 86.71 (19.06) mg/dL; and TG, 87.86 (34.40) mg/dL (Table 1).

In each group, the value of arterial blood pressure (BP) was also assessed. The average values of both systolic and diastolic BP, 128.3 (15.7) mmHg and 77.4 (10.4) mmHg, respectively, were significantly higher in the study group (*p* = 0.0001 and *p* < 0.0001) than in the control group, where they were as follows: systolic BP, 109.9 (13.2) mmHg; diastolic BP, 64.3 (9.0) mmHg. The percentages of normal blood pressure in the study group and control group were 33.9% vs. 78.6%; too high, 50.8% vs. 0.0%; and high normal, 15.3% vs. 21.4%, respectively. The BP distributions were statistically significantly different between the groups (*p* = 0.0018) (Table 2).

In terms of inflammatory markers, significantly higher levels of interleukin 6 (IL-6) were demonstrated in the study group compared to the control group (6.17 ± 11.78 vs. 0.57 ± 0.58 pg/mL; *p* = 0.0004). Interleukin 10 (IL-10) and tumor necrosis factor alpha (TNF-α) levels did not differ significantly between study groups (*p* = 0.1693 and *p* = 0.8979, respectively), whereas vascular endothelial growth factor (VEGF-a) levels were significantly higher in the study group (231.85 ± 179.03 pg/mL) compared to the control group (170.11 ± 202.05 pg/mL; *p* = 0.0471). With regard to markers, insulin concentration was significantly increased in the study group (711.50 ± 737.31 pg/mL) compared to the control group (277.83 ± 111.89 pg/mL; *p* = 0.0022); a similar relationship was observed for leptin, the level of which in patients in the study group was 16,277.19 ± 11,455.20 pg/mL, and in the control group was 3718.39 ± 5465.50 pg/mL (*p* < 0.0001). In turn, adiponectin showed the opposite trend—its concentration was significantly lower in the study group (40,614.36 ± 23,367.78 pg/mL) compared to in the control group (102,666.67 ± 68,493.83 pg/mL; *p* = 0.0001). No significant differences in ghrelin concentration were found between the patients from the control and study groups (*p* = 0.0775). Similarly, resistin levels were higher in the study group (55.54 ± 47.35 pg/mL) compared to in the control group (36.22 ± 18.89 pg/mL); however, this difference did not reach statistical significance (*p* = 0.0633) (Table 3).

Analysis of correlations between the levels of the biomarkers studied and systolic and diastolic blood pressure showed significant statistical dependencies. In the case of systolic blood pressure (SBP), a significant positive correlation was found with the levels of IL-6 (R = 0.32, *p* = 0.0064), insulin (R = 0.25, *p* = 0.0326), leptin (R = 0.25, *p* = 0.0355), and resistin (R = 0.26, *p* = 0.0271). In addition, age (R = 0.37; *p* = 0.0012) and BMI (R = 0.47; *p* = 0.0000) also showed a strong positive correlation with SBP. On the other hand, ghrelin level showed a significant negative correlation with SBP (R = −0.31; *p* = 0.0089). A significant negative correlation was also noted for HDL levels (R = −0.27; *p* = 0.0189), suggesting its potential protective role in blood pressure regulation. VEGF-a did not show a significant correlation with SBP (*p* = 0.1088), similar to IL-10, TNF-α, glucose, total cholesterol, and LDL (*p* > 0.05). In relation to diastolic blood pressure (DBP), significant positive correlations were observed for VEGF-a (R = 0.27; *p* = 0.0203), insulin (R = 0.31; *p* = 0.0091), and leptin (R = 0.38; *p* = 0.0012). A positive correlation was also found for age (R = 0.27; *p* = 0.0192) and BMI (R = 0.34; *p* = 0.0028). In turn, ghrelin levels showed a significant negative correlation with DBP (R = −0.31; *p* = 0.0093). Adiponectin (R = −0.25; *p* = 0.0347) and HDL (R = −0.22; *p* = 0.0610) levels also correlated negatively with DBP, but in the case of HDL, the result did not reach the level of statistical significance. The correlations of IL-6 and resistin with DBP did not reach the level of statistical significance (*p* > 0.05) (Table 4).

Analysis of the area under the ROC curve (AUC) values showed that the highest diagnostic ability of the metabolites analyzed in the study was demonstrated by leptin (AUC = 0.72, 95% CI: 0.59–0.85, *p* = 0.0011), IL-6 (AUC = 0.69, 95% CI: 0.55–0.83, *p* = 0.0066), and VEGF-a (AUC = 0.66, 95% CI: 0.51–0.81, *p* = 0.0326). These values indicate a statistically significant ability of these biomarkers to differentiate the studied groups.

The sensitivity and specificity of individual markers were varied. BMI was characterized by 100% sensitivity but relatively low specificity (41.9%). Leptin (96.7%) and VEGF-a (83.3%) also showed high sensitivity, with specificities of 41.4% and 65.5%, respectively. IL-6 had moderate sensitivity (66.7%) and specificity (72.4%).

The cut-off points for significant markers were as follows: 1 pg/mL for IL-6, 130.37 pg/mL for VEGF-a, and 5053 pg/mL for leptin, which are the borderline values that may have potential clinical use. The remaining analyzed biomarkers, including IL-10, TNF-α, ghrelin, insulin, adiponectin, resistin, glucose, cholesterol, LDL, and HDL, did not reach statistical significance (*p* > 0.05), which suggests their limited diagnostic value in the study group (Table 5).

## 3. Discussion

With the growing epidemic of obesity in people under 18, the risk of AH in this age group is increasing. Higher BP values may result from several factors, such as insufficient angiogenesis for the increasing body surface area, pressure of perivascular fat tissue on blood vessels, or impaired balance of the renin–angiotensin–aldosterone system (RAAs), which regulates blood pressure by controlling water and sodium retention in the kidneys [26,27,28,29,30].

Our study showed that the basic obesity indicator—BMI—was significantly positively correlated with higher systolic and diastolic blood pressure values in children and adolescents. Moreover, after using the receiver operating curve (ROC) analysis, its sensitivity in relation to blood pressure was 100%. Interestingly, the cut-off point for BMI was 29 kg/m^2^, which is the borderline value between overweight and obesity of the first degree in adults. It is worth emphasizing that as many as 50.8% of children with obesity had blood pressure values > 95 cc RR, while in the control group the percentage of such children was 0%.

Analyzing the study and control groups in terms of lipid and carbohydrate profiles, characteristic metabolic changes were observed in children with obesity. They included lower HDL levels and higher LDL and triglyceride values compared to the control group. The negative correlation of HDL with SBP (*p* = 0.0189) is particularly significant, confirming its potential protective function for the vascular system, which is described in the literature. Cho et al. in 2021, in their study, presented results that proved the rapid decline in HDL-C levels during puberty was associated with increased blood pressure and dyslipidemia in Korean boys, emphasizing the dynamic interdependence between lipid profiles and vascular parameters during puberty [31]. The lack of significant correlations between total cholesterol and LDL values and blood pressure may be due to the fact that these are less-dynamic parameters and respond more slowly to metabolic changes than adipokines. An interesting observation is the fasting glucose level, which was paradoxically lower in the study group, which may be related to compensatory hyperinsulinemia, which is sometimes observed in the early stages of insulin resistance—a disorder that accompanies obesity. Confirmation of these assumptions should be based on the increased insulin level, which was noted in the study group included in the above study. In patients with obesity, insulin levels were not only significantly higher than in the control group, but insulin also showed a positive, statistically significant effect on both systolic and diastolic blood pressure. This situation may result from the long-term effects of insulin and its modeling of blood pressure over the course of several or a dozen years, which is mentioned in the 2016 paper by de Giorgis et al. The researchers conducted a study on adolescent patients in which they confirmed that both fasting insulin levels and the HOMA-IR index in childhood were positively associated with blood pressure values in adolescence [32]. Considering that each of the above-mentioned parameters significantly differed in their levels in the study and control groups, but was not a leading predictor of hypertension on its own, consideration should be given to developing new indicators that would be based on the ratio of individual parameters and could reliably reflect the risk of developing hypertension.

Of particular note are the adipokines included in the above study, whose special role in the development of hypertension was demonstrated by the statistical analyses performed. Among the adipokines tested, leptin, a hormone produced by adipocytes, has the highest sensitivity and statistical ability in relation to hypertension in children suffering from obesity. Additionally, leptin showed a positive correlation with both systolic and diastolic blood pressure, and its level was significantly higher in the study group than in the control group. The ROC analysis noted that the AUC (95% Cl), sensitivity, and specificity of leptin were similar to the values of the parameters given for BMI. An interesting observation was noted by Ahiante et al., who found a positive correlation between leptin and 24-h diastolic blood pressure in white men (*p* = 0.006), while not finding such a correlation in women or black men. This may indicate differences in the functioning of the autonomic system and hormonal factors between representatives of other sexes or ethnic groups [33].

In contrast to the results of our study, there is a 2020 paper by Varda et al. [34], in which no significant relationship was found between hypertension in children with obesity and serum leptin concentration. However, despite the lack of significant associations between leptin and cardiovascular risk, the researchers showed a negative correlation between ghrelin and adiponectin and systolic blood pressure in patients with obesity. According to their results, the most promising marker of cardiovascular disease should be ghrelin, the concentration of which decreases with increasing adipose tissue and increasing systolic blood pressure [34]. A negative correlation between ghrelin and systolic hypertension was also noted in a study of 387 women in China [35].

This is partly consistent with our study, in which we also noted a significant negative correlation of ghrelin with diastolic and systolic blood pressure in children and adolescents affected by obesity, which indicates a potential protective role in the context of hypertension in the course of obesity. We also found a statistically significant negative correlation between adiponectin and diastolic blood pressure. The role of reduced adiponectin levels in the development of hypertension was highlighted, among others, in a study published in 2016 by Peri-Okonny et al. on a large group of adults (1233 participants) over a 7-year period. The authors described the antihypertensive role of adiponectin, regardless of the presence or absence of obesity in patients [36]. Moreover, in 2022, Wu et al. [37] published a paper in which it was noted that not only adiponectin, but also resistin, has an impact on the increase in blood pressure in the course of obesity. The researchers developed an adiponectin–resistin (AR) index, which seemed to be more strongly associated with the increase in blood pressure values than isolated levels of the adipokines [37]. The relationship between adiponectin and resistin may be due to the co-expression of these metabolites from adipocytes, but few studies have been conducted on the AR index [37,38].

The influence of resistin itself on blood pressure values is also widely described in the literature [37,38,39,40,41,42,43,44]. In our study, despite the fact that resistin does not lead in terms of predictive capabilities in relation to the risk of hypertension, we noted its significant effect on systolic blood pressure. Interestingly, according to our study, resistin statistically significantly positively correlates with high systolic blood pressure values, while in the context of diastolic blood pressure values, its correlation, although also positive, is not statistically significant (*p* = 0.061). Additionally, this adipokine showed greater sensitivity (69%) to high blood pressure values than leptin and BMI. Such results are not reflected in the literature, which remains in great divergence on the subject of the role of resistin in the genesis of hypertension. Correlations between resistin in hypertension in the course of obesity were noted by Wu et al. in 2022, but according to their results, this correlation should be negative [37]. Different results were obtained by Ding et al., 2018 [45] and Musialik et al., 2022 [46] who did not find any differences in resistin levels between patients with and without hypertension in both adult and pediatric patients. However, Musialik et al. pointed out that the discrepancy between the estimated correlations of resistin with high blood pressure values may be due to the failure to take into account specific genetic variants of resistin associated with the expression level of this adipokine [46].

Our study also included two proteins—VEGF, which belongs to the family of growth factors and is a signaling protein, key in the process of angiogenesis, and IL-6, which is a cytokine, i.e., a regulatory protein involved in the inflammatory and immune response of the body. Both proteins had significantly higher levels in the group of children with obesity than in the control group. Additionally, both VEGF and IL-6, right after leptin, showed the highest statistical ability in relation to higher blood pressure values in people with obesity. While IL-6 showed a positive correlation and moderate sensitivity in relation to systolic blood pressure values, an increase in VEGF level was associated with an increase in diastolic blood pressure values and showed high sensitivity in our study. It is also worth noting that both IL-6 and VEGF showed higher specificity for blood pressure than BMI or leptin. In the current literature, although few studies focus on the role of the above proteins in the context of hypertension in the course of obesity, the available studies also see the potential of VEGF and IL-6 as markers of the risk of hypertension [47,48,49,50,51]. Both Gondim et al. and Bochar et al. described a possible effect of IL-6 on elevated BP values by increasing inflammation and endothelial dysfunction, which may promote increased vascular resistance [52]. In turn, observations regarding the role of VEGF show that this protein, in addition to its angiogenic functions, supports the regulation of blood pressure by influencing the integrity of the endothelium [50].

## 4. Materials and Methods

### 4.1. Characteristics of Patients Included in the Study

The study and control groups were created based on cooperation with the Department of Pediatric Endocrinology and Diabetology at the University Children’s Hospital in Lublin, based on a previously concluded agreement.

The study included a group of 78 patients. The research group consisted of 60 patients with obesity, and the control group consisted of 18 patients with normal body weight.

Patients were referred for the study by specialists and residents during specialization in pediatrics, diabetology, and/or endocrinology from the Department of Pediatric Endocrinology and Diabetology at the University Children’s Hospital. The study included anthropometric measurements such as height measurement using a stadiometer and weight measurement using a medical scale, as well as an assessment of the patients’ sexual maturity levels according to the Tanner scale. Each participant had their blood pressure assessed according to the recommendations of the Polish Society of Hypertension [53]. Blood pressure was measured in resting conditions, after the child had rested for at least 5 min in a sitting position. A sphygmomanometric device (Heine Gamma GP, Heine, Gilching, Germany) with a cuff in sizes appropriate for children was used for the measurement. The cuff was selected individually for each child, so that the width of the cuff was approximately 40% of the patient’s arm circumference and its length covered 80–100% of the arm circumference. Measurements were taken on the right arm, held at the level of the heart. Three consecutive measurements were taken with an interval of 1–2 min. The arithmetic mean of the second and third measurements was used for statistical purposes. The obtained value was referred to the percentile charts for blood pressure in relation to height, age, and gender. Values above 95 pc were defined as elevated BP based on the guidelines of the Polish Society of Pediatric Nephrology (PTNFD), which are consistent with European standards [53,54,55] (Table 1 and Table 2).

The criterion for qualifying for the study group was body mass index (BMI) calculated on the basis of anthropometric measurements, which was then applied to the OLA and OLAF centile charts [56,57], i.e., to current samples representative of the Polish population of children and adolescents aged 3–18. The obtained results were referred to the World Health Organization (WHO) guidelines for the diagnosis of obesity published in 2022:

- Children under 5 years of age—obesity is a body mass-to-height ratio greater than 3 standard deviations above the median of the WHO Child Growth Standards;
- Children aged 5 to 19 years—obesity is a body mass-to-height ratio greater than 2 standard deviations above the median of the WHO Growth Reference.

According to the available literature, it is possible to reference the Polish BMI centile charts with the BMI centile charts developed by the WHO. Such action may lead to underestimation of the prevalence of being overweight; however, in this study we did not distinguish this intermediate group between normal BMI and BMI indicating obesity; therefore, we decided to use such a reference [7,56,57,58].

The exclusion criteria for the study were the lack of consent from patients or parents of patients, the occurrence of congenital genetic diseases causing metabolic syndrome from early childhood, taking medications affecting glucose–lipid metabolism, current pregnancy, or being within 6 months of a previous pregnancy (information based on an interview with the patient).

The study received approval from the Bioethics Committee No. (KE-0254/25/2020) at the Medical University of Lublin.

### 4.2. Material for the Study

Blood was collected from both the control group and the study group, which consisted of patients after a night’s rest and 12 h after the most recent meal.

Peripheral blood in the amount of 10 mL was collected in a tube for clotting. To obtain serum, each collected blood sample was immediately centrifuged for 10 min at °C at 300 rpm. Then, to obtain plasma, each sample was centrifuged at room temperature for 10 min at 700 rpm. In the next step, 400 µL of the centrifuged serum was pipetted into 6 Eppendorf TM tubes with a capacity of 1.5 mL, and 400 µL of the centrifuged plasma into 1 Eppendorf TM tube with a capacity of 1.5 mL. The material obtained in this way was frozen at −20 °C. The serum samples from each patient were transported to the Department of Immunobiology and Environmental Microbiology at the Medical University of Gdansk for further testing for the presence of cytokines and adipokines.

### 4.3. Testing of Basic Parameters in Blood Serum

The level of glucose exponents and the lipid profile (total cholesterol, triglycerides, high-density lipoprotein, low-density lipoprotein) were determined in the Medical Analysis Laboratory at the University Children’s Hospital in Lublin. Roche technology was used to determine the above parameters according to standard procedures: glucose was determined using the hexokinase method (ELECYC-Cobasc311), while the lipid profile was determined using the enzymatic–colorimetric method (ELECYC-CobasIntegra 400 plus, and HDL-ELECYC-Cobasc311, Roche Diagnostics, Basel, Switzerland).

### 4.4. Analysis of Cytokines and Adipokines

Serum metabolites were determined accordingly: cytokines interleukin 10 (IL-10), IL-6, and VEGF-a by the MILLIPLEX^®^ Human Cytokine/Chemokine/Growth Factor Panel A Kit (HCYTA-60K-04, R&D Systems, Minneapolis, MN, USA); ghrelin, insulin, and leptin by the MILLIPLEX^®^ Human Metabolic Hormone Panel V3 (HMH3-34K-03, R&D Systems, USA); and adiponectin and resistin by the MILLIPLEX^®^ Human Adipokine Magnetic Bead Panel 1—Endocrine Multiplex Assay (HADK1MAG-61K-02, R&D Systems, USA). The kit protocol was followed to collect and prepare serum samples from patients in the control and study groups. In accordance with the manufacturer’s instructions, the procedure was executed as previously described. To summarize, serum samples were diluted in accordance with the protocol instructions and incubated with color-coded magnetic beads either overnight (HADK1MAG-61K and HMH3-34K-03) at 4 °C or for 2 h (HCYTA-60K-04) at room temperature. Subsequently, the plates were washed, and detection antibodies were added for 1 h. Streptavidin-phycoerytrin was then added, and the plates were incubated for 30 min. After another wash, the fluorescence intensity (MFI) was measured on a MAGPIX^®^ System (Luminex^®^ Corporation, Austin, TX, USA), and the data was analyzed using the MILLIPLEXC Analyst software v5.1 [59,60].

### 4.5. Statistical Analysis of the Obtained Results

All statistical analyses were performed using the STATISTICA software package (StatSoft Inc.; version 12.0, Tulsa, OK, USA) and Microsoft Excel. Quantitative variables were summarized using descriptive statistics, including mean, standard deviation, median, minimum and maximum values (range), and 95% CI (confidence interval). Qualitative variables were expressed as absolute frequencies and percentages. Normality of distribution of continuous variables was assessed using Shapiro–Wilk test, and homogeneity of variance was assessed using Levene’s test (Brown–Forsythe modification). Student’s *t*-test was used for comparisons between two independent groups when parametric assumptions were met. Student’s *t*-test was used in cases of unequal variances. The Mann–Whitney U test was used when assumptions of normality were not met or for ordinal data. Comparisons involving more than two independent groups were performed using one-way analysis of variance (ANOVA) or the Kruskal–Wallis test, as appropriate. When statistically significant differences were detected, post hoc analyses were performed using Tukey’s test after ANOVA or Dunn’s test after the Kruskal–Wallis test. Student’s *t*-test for dependent samples was used to analyze two related variables, and the Wilcoxon signed-rank test was used when the assumptions of the parametric test were not met or for variables measured on an ordinal scale. Differences between more than two related variables were assessed using repeated-measures analysis of variance, and the Friedman test was used when its assumptions were not met or for ordinal data. Relationships between qualitative variables were analyzed using the chi-square test for independence, applying Yates’ correction for cell numbers below 10; Fisher’s exact test was used if Cochran’s conditions were not met. Correlation analysis was used to assess the existence, strength, and direction of relationships between quantitative variables, calculating Pearson or Spearman correlation coefficients, depending on the nature of the data. A statistical significance level of *p* = 0.05 was used for all analyses. To determine the predictive value of selected clinical and biochemical parameters in relation to the risk of developing hypertension, ROC curve analysis was performed, including assessment of the area under the curve (AUC), sensitivity and specificity, and determination of the optimal cut-off point. The Youden index was used to estimate the cut-off point value [61]. The nonparametric DeLong method was used to calculate the statistical value for AUC [62]. DeLong’s test was also used to compare ROC analyses. When adjustment of ROC curves with different orientations was required, a non-parametric bootstrap method with 2000 resamples was applied [63]. A *p*-value of 0.05 was set as the threshold for statistical significance.

## 5. Conclusions

The results of the analyses indicate that a number of adipokines and metabolic hormones play an important role in the pathogenesis of hypertension in children and adolescents with obesity. The above study showed that leptin, IL-6, and VEGF-a have high statistical ability in relation to high RR values in obesity in children and adolescents. This means that their levels should be taken into account in clinical practice to predict the development of hypertension in children and adolescents with obesity.

Of the given group, leptin and VEGF are characterized by the highest sensitivity, so the change in their serum levels will be visible as the fastest in people at high risk of hypertension. Additionally, leptin levels correlate positively with both increased systolic and diastolic blood pressure, while VEGF shows a positive correlation only with increased systolic blood pressure. Therefore, leptin levels may best reflect disorders in the cardiovascular system. Although IL-6 has statistical power equal to leptin and VEGF, its sensitivity and specificity are moderate, so the reliability of its levels in clinical practice in the context of the risk of increased blood pressure is limited.

Although ghrelin was not ranked as the strongest predictor of hypertension, due to its negative correlation with systolic and diastolic pressure, it seems to play a protective role and may be used in the future to exclude the risk of hypertension or to prevent cardiovascular diseases.

The role of adiponectin and resistin in the development of hypertension remains unclear and requires broader studies.

Insulin, through its effect on sodium retention and activation of the sympathetic nervous system, may also indirectly increase blood pressure, but broader studies focusing on the study of the correlation of hyperinsulinemia, which occurs in obesity, with the risk of hypertension in children and adolescents are needed.

The study results emphasize the need to monitor BMI and metabolic and inflammatory biomarkers in children and adolescents for early detection of hypertension risk. Further research may help identify the mechanisms underlying these associations and develop effective preventive and therapeutic strategies.

## Figures and Tables

**Table 1:** Baseline characteristics of the study and control groups, including sex, age, and selected clinical parameters (glucose, insulin, total cholesterol, LDL, HDL, and triglycerides).

	Study Group (n = 60)	Control Group (n = 18)	*p*-Value
**Gender**			0.8038 ^1^
Boy/man	28 (46.7%)	9 (50.0%)	
Girl/woman	32 (53.3%)	9 (50.0%)	
**Pubertal phase according to Tanner scale**			0.0351 ^1^
1	2 (3.3%)	3 (21.4%)	
2	4 (6.7%)	1 (7.1%)	
3	11 (18.3%)	3 (21.4%)	
4	12 (20.0%)	5 (35.7%)	
5	31 (51.7%)	2 (14.3%)	
**Age [years]**			0.0570 ^3^
Average value (SD)	14.6 (1.5)	12.4 (3.8)	
Range	12.0–17.1	6.7–17.9	
Median (IRQ)	14.6 (2.7)	13.2 (6.9)	
95%CI	[14.2; 14.9]	[10.5; 14.3]	
**BMI [kg/m^2^]**			<0.0001 ^1^
Average value (SD)	35.7 (4.6)	17.7 (2.6)	
Range	23.9–49.9	14.7–23.2	
Median (IRQ)	35.1 (6.4)	17.2 (3.6)	
95%CI	[34.5; 36.9]	[16.4; 18.9]	
**Glucose [mg/dL]**			<0.0001 ^2^
Average value (SD)	80.99 (8.13)	93.29 (5.50)	
Range	64.00–98.00	84.00–100.93	
Median (IRQ)	80.28 (13.14)	92.68 (9.69)	
95%CI	[78.89; 83.09]	[90.55; 96.02]	
**Insulin [pg/mL]**			0.0022
Average value (SD)	711.50 (737.31)	277.83 (111.89)	
Range	186.37–3 991.00	186.37–566.25	
Median (IRQ)	427.99 (623.26)	237.40 (159.54)	
95%CI	[517.63; 905.36]	[222.18; 333.47]	
**Cholesterol [mg/dL]**			0.9121 ^3^
Average value (SD)	160.22 (32.90)	158.29 (21.51)	
Range	103.00–272.00	119.00–197.00	
Median (IRQ)	157.00 (36.98)	157.00 (32.00)	
95%CI	[151.72; 168.72]	[145.87; 170.70]	
**LDL [mg/dL]**			0.0064 ^2^
Average value (SD)	110.17 (29.79)	86.71 (19.06)	
Range	57.00–179.00	57.00–114.00	
Median (IRQ)	106.00 (44.50)	89.00 (25.00)	
95%CI	[102.47; 117.86]	[75.71; 97.72]	
**HDL [mg/dL]**			0.0002 ^3^
Average value (SD)	40.42 (10.72)	53.78 (11.86)	
Range	24.00–92.00	38.90–71.90	
Median (IRQ)	38.80 (12.60)	53.90 (19.10)	
95%CI	[37.65; 43.19]	[46.93; 60.63]	
**TG [mg/dL]**			0.0378 ^3^
Average value (SD)	111.08 (46.62)	87.86 (34.40)	
Range	37.00–271.00	56.00–183.00	
Median (IRQ)	97.50 (52.50)	76.50 (34.00)	
95%CI	[99.04; 123.13]	[68.00; 107.72]	

^1^ Chi-square, ^2^ t-Student; ^3^ U Mann–Whitney. Abbreviations: BMI—body mass index [kg/m^2^]; LDL—low-density lipoproteins; HDL—high-density lipoprotein; TG—triglycerides.

**Table 2:** Comparison of the study and control groups in terms of systolic and diastolic blood pressure values.

	Study Group (n = 60)	Control Group (n = 18)	*p*-Value
**BP systolic [mmHg]**			0.0001 ^2^
Average value (SD)	128.3 (15.7)	109.9 (13.2)	
Range	98.0–185.0	85.0–130.0	
Median (IRQ)	127.0 (19.0)	110.0 (20.0)	
95%CI	[124.2; 132.4]	[102.3; 117.6]	
**BP diastolic [mmHg]**			<0.0001 ^2^
Average value (SD)	77.4 (10.4)	64.3 (9.0)	
Range	55.0–105.0	50.0–75.0	
Median (IRQ)	76.0 (15.0)	65.0 (15.0)	
95%CI	[74.7; 80.1]	[59.1; 69.5]	
**BP [mmHg]**			0.0018 ^1^
Normal	20 (33.9%)	11 (78.6%)	
High	30 (50.8%)	0 (0.0%)	
Elevated	9 (15.3%)	3 (21.4%)	

^1^ Chi-square; ^2^ t-Student. Abbreviation: BP—blood pressure.

**Table 3:** Differences in metabolite profiles between the study and control groups.

	Study Group (n = 60)	Control Group (n = 18)	*p*-Value
**IL-6 [pg/mL]**			0.0004
Average value (SD)	6.17 (11.78)	0.57 (0.58)	
Range	0.23–55.88	0.23–2.53	
Median (IRQ)	1.08 (4.08)	0.31 (0.35)	
95%CI	[3.07; 9.27]	[0.28; 0.86]	
**IL-10 [pg/mL]**			0.1693
Average value (SD)	3.59 (4.38)	4.22 (3.85)	
Range	0.88–22.26	0.88–16.58	
Median (IRQ)	1.72 (3.19)	3.58 (3.18)	
95%CI	[2.43; 4.74]	[2.31; 6.14]	
**TNF-α [pg/mL]**			0.8979
Average value (SD)	19.69 (9.61)	19.39 (8.37)	
Range	3.56–46.51	6.29–33.37	
Median (IRQ)	16.91 (11.67)	17.39 (14.34)	
95%CI	[17.16; 22.21]	[15.23; 23.55]	
**VEGF-a [pg/mL]**			0.0471
Average value (SD)	231.85 (179.03)	170.11 (202.05)	
Range	23.10–761.69	15.46–752.13	
Median (IRQ)	177.60 (214.56)	60.13 (218.38)	
95%CI	[184.77; 278.92]	[69.64; 270.59]	
**Ghrelin [pg/mL]**			0.0775
Average value (SD)	9.00 (0.00)	11.79 (7.83)	
Range	9.00–9.00	9.00–40.66	
Median (IRQ)	9.00 (0.00)	9.00 (1.20)	
95%CI	[0.00; 0.00]	[7.90; 15.69]	
**Leptin [pg/mL]**			<0.0001
Average value (SD)	16,277.19 (11,455.20)	3718.39 (5465.50)	
Range	1294.00–52,943.00	149.10–19,567.00	
Median (IRQ)	14,709.50 (14,870.00)	1197.00 (5262.97)	
95%CI	[13,265.20; 19,289.18]	[1000.45; 6436.32]	
**Adiponectin [pg/mL]**			0.0001
Average value (SD)	40,614.36 (23,367.78)	102,666.67 (68,493.83)	
Range	9332.00–124,224.00	16,693.00–262,131.00	
Median (IRQ)	34,146.50 (22,753.00)	87,891.50 (63,555.00)	
95%CI	[34,470.12; 46,758.61]	[68,605.48; 136,727.85]	
**Resistin [pg/mL]**			0.0633
Average value (SD)	55.54 (47.35)	36.22 (18.89)	
Range	20.80–342.02	10.69–85.28	
Median (IRQ)	45.68 (38.89)	33.34 (25.15)	
95%CI	[43.09; 67.99]	[26.83; 45.61]	

U Mann–Whitney. Abbreviations: IL-6—interleukin 6; IL-10—interleukin 10; TNF-α—tumor necrosis factor alpha; VEGF—vascular endothelial growth factor.

**Table 4:** Correlation analysis—searching for predictors of systolic and diastolic blood pressure in children and adolescents.

	Systolic Blood Pressure		Diastolic Blood Pressure	
	R	*p*-Value	R	*p*-Value
Age	0.37	0.0012	0.27	0.0192
BMI	0.47	0.0000	0.34	0.0028
IL-6	0.32	0.0064	0.16	0.1799
IL-10	−0.05	0.6523	0.02	0.8883
TNF-α	−0.05	0.6846	−0.04	0.7561
VEGF-a	0.19	0.1088	0.27	0.0203
Ghrelin	−0.31	0.0089	−0.31	0.0093
Insulin	0.25	0.0326	0.31	0.0091
Leptin	0.25	0.0355	0.38	0.0012
Adiponectin	−0.17	0.1500	−0.25	0.0347
Resistin	0.26	0.0271	0.22	0.0672
Glucose	−0.06	0.6052	−0.01	0.9116
Cholesterol	−0.06	0.6425	−0.05	0.6929
LDL	0.06	0.6181	0.12	0.2927
HDL	−0.27	0.0189	−0.22	0.0610

Abbreviations: BMI—body mass index [kg/m^2^]; IL-6—interleukin 6; IL-10—interleukin 10; TNF-α—tumor necrosis factor alpha; VEGF—vascular endothelial growth factor; LDL—low-density lipoproteins; HDL—high-density lipoprotein.

**Table 5:** ROC analysis—searching for predictors of systolic and diastolic blood pressure in children and adolescents.

	AUC (95%CI)	*p*-Value	Sensitivity	Specificity	Cut-Off Point	PPV	NPV
Age	0.50 (0.35–0.65)	0.9886	93.3%	22.6%	12.11	53.8%	77.8%
BMI	0.73 (0.60–0.85)	0.0005	100.0%	41.9%	29	62.5%	100.0%
IL-6	0.69 (0.55–0.83)	0.0066	66.7%	72.4%	1	71.4%	67.7%
IL-10	0.53 (0.37–0.68)	0.7433	80.0%	34.5%	1.38	55.8%	62.5%
TNF-α	0.50 (0.35–0.65)	0.9700	13.3%	100.0%	36.01	100.0%	52.7%
VEGF-a	0.66 (0.51–0.81)	0.0326	83.3%	65.5%	130.37	71.4%	79.2%
Ghrelin	0.55 (0.40–0.70)	0.4934	100.0%	10.3%	9	53.6%	100.0%
Insulin	0.57 (0.42–0.71)	0.3856	30.0%	89.7%	861.41	75.0%	55.3%
Leptin	0.72 (0.59–0.85)	0.0011	96.7%	41.4%	5053	63.0%	92.3%
Adiponectin	0.59 (0.44–0.74)	0.2494	86.7%	41.4%	63,835	60.5%	75.0%
Resistin	0.57 (0.42–0.72)	0.3393	50.0%	69.0%	48.71	62.5%	57.1%
Glucose	0.57 (0.42–0.71)	0.3724	53.3%	64.5%	82	59.3%	58.8%
Cholesterol	0.55 (0.41–0.70)	0.4828	93.3%	22.6%	130	53.8%	77.8%
LDL	0.63 (0.49–0.77)	0.0701	96.7%	25.8%	75	55.8%	88.9%
HDL	0.60 (0.45–0.74)	0.1952	70.0%	51.6%	44	58.3%	64.0%

Abbreviations: BMI—body mass index [kg/m^2^]; IL-6—interleukin 6; IL-10—interleukin 10; TNF-α—tumor necrosis factor alpha; VEGF—vascular endothelial growth factor; LDL—low-density lipoproteins; HDL—high-density lipoprotein.

## Data Availability

The original contributions presented in this study are included in the article. Further inquiries can be directed to the corresponding author.
